# The Synthesis of Low-Viscosity Organotin-Free Moisture-Curable Silane-Terminated Poly(Urethane-Urea)s

**DOI:** 10.3390/polym10070781

**Published:** 2018-07-16

**Authors:** Chen Tan, Viivi Luona, Teija Tirri, Carl-Eric Wilen

**Affiliations:** Laboratory of Polymer Technology and Center for Functional Materials, Faculty of Science and Engineering/Chemical Engineering, Åbo Akademi University, Biskopsgatan 8, 20500 Turku, Finland; ctan@abo.fi (C.T.); vluona@abo.fi (V.L.); teija.tirri@abo.fi (T.T.)

**Keywords:** moisture-curable silane-terminated poly(urethane-urea)s (SPUR), organotin-free catalyst, silane end-capped, physicochemical properties

## Abstract

This work explores the possibility of synthesizing moisture-curable silane-terminated poly(urethane-urea)s (SPURs) of low viscosity. First, NCO-terminated urethane prepolymers were prepared, followed by silane end-capping. The impact of polyol molecular weight and the ratio of isocyanate to polyol (NCO/OH) on viscosity and the properties of SPUR were examined. As alternatives to the organotin catalysts traditionally used for the polyurethane synthesis and curing processes, bismuth carboxylate catalysts were evaluated. In addition, the effect of organofunctional groups in the aminosilane structure (R1–NH–R2–Si(OR3)_3_), i.e., R1 (alkyl, aryl or trimethoxysilyl-propyl), the spacer R2 (α or γ) and alkyl group R3 (methyl or ethyl), was examined. The chemical and physical structures of the SPUR were investigated by nuclear magnetic resonance spectroscopy (NMR), Fourier transform infrared spectroscopy (FT-IR) and the mechanical properties were evaluated by tensile tests. The results reveal that silane-terminated, moisture-curable polyurethanes can be successfully synthesized and cured with bismuth carboxylate catalysts. SPUR exhibiting low viscosity, with adequate tensile strength and elongation can be prepared using environmentally benign bismuth carboxylate catalyst having a high metal content of 19%–21%, by utilizing secondary aminosilane end-cappers and an optimal combination of the polyol molecular weight and NCO/OH ratio.

## 1. Introduction

Polyurethanes (PURs) have a widespread application range due to their ease of use and because their mechanical, thermal, and chemical properties can be tuned by rational design of the molecular chain structure. However, in recent years, traditional PURs that contain free isocyanate groups, including isocyanate-terminated urethane prepolymers, have been under scrutiny due to the occupational health concerns related to respiratory and skin exposures [1,2,3,4]. Also, the organotin catalysts used for their production and curing have potential harmful environmental effects [5,6,7,8].

Due to the aforementioned circumstances, there is urgent need to further refine and develop polyurethanes by utilizing more sustainable chemistries that are free of terminal isocyanate groups and that use more environmentally benign catalysts than dibutyltin dilaurate (DBTL) to accelerate their preparation and/or curing processes.

Towards this end, a number of groups have started to develop moisture-curable silane-terminated poly(urethane-urea)s (SPURs) that consist of a polyurethane backbone terminated by moisture-curable alkoxysilanes [9,10,11,12,13]. Since SPUR contains no isocyanate residues and the curing process of SPUR does not generate carbon dioxide, some deficits inherently present in conventional PURs can also be circumvented. In addition to these benefits, SPUR also possesses hybrid properties of polyurethanes and silicones.

**Figure 1:**
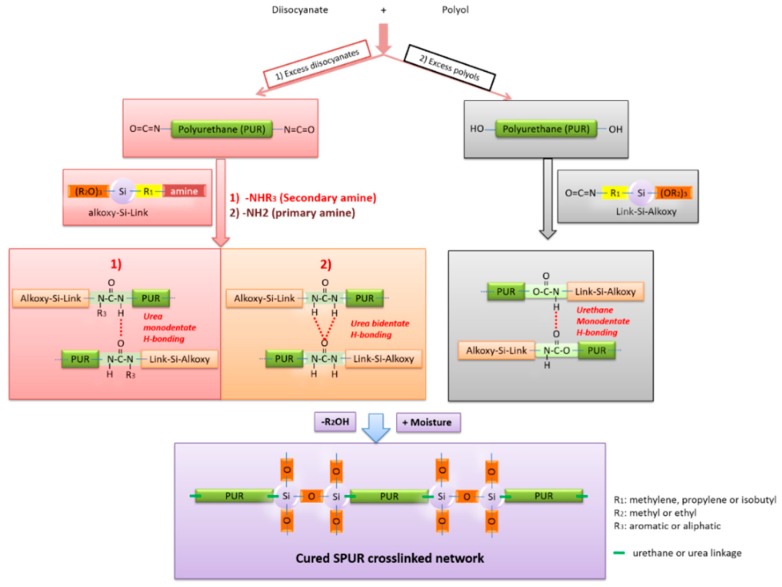
Two general routes for SPUR synthesis.

In general, there are two synthetic routes to SPURs, which are presented in Figure 1: (1) a NCO-terminated urethane prepolymer that is reacted with aminosilane; and (2) an OH-terminated urethane prepolymer that is reacted with isocyanate silane. Route 1 yields SPUR with a high viscosity as a result of substantial content of hard segments and rigid urea linkages by aminosilane end-capping. The attractiveness of route 1 is that the urea linkages give better mechanical and physical performance than those based on conventional urethane linkages. On the other hand, the high viscosity ranging from 50,000 to 200,000 mPa·s makes the wetting of surfaces worse. To some extent, the increase in viscosity can be reduced by using secondary aminoalkoxysilanes that yield less ordered monodentate urea structures (Figure 1(1)) instead of primary ones, which result in a highly ordered bidentate urea structures (Figure 1(2)) [12].

During application, the produced SPUR cures by atmospheric moisture to yield highly stable siloxane crosslinked network structures. Thus, the formed hybrid system of SPUR combines the unique and beneficial features of inorganic, elastic silicone with organic, tough polyurethane, whereby formulations exhibiting water resistance, heat resistance, high tensile strength and elongation can be obtained [10,11,14,15].

Several studies have been carried out on conventional polyurethanes, and thereby insights have been gained on their structure-property relationships. PUR properties can be tuned over a wide range by adjusting the molecular weight and the composition of polymer main chains as well as the extent of intra-and intermolecular hydrogen bonding [16,17,18,19,20,21]. However, to the best of our knowledge, the effect of organosilane type and bismuth-based catalysts on the properties and performance of SPUR has not been systematically studied.

The purpose of this work was to explore the possibility of synthesizing moisture-curable silane-terminated poly(urethane-urea)s of low-viscosity. As promising alternatives to organotin catalysts, bismuth carboxylate-based catalysts were evaluated. In addition, the effect of using different organofunctional silanes R1–NH–R2–Si(OR3)_3_ in terms of the nature of amino substituent R1 (alkyl, aryl or trimethoxysilyl-propyl), the spacer R2 (α or γ) and alkyl group R3 (methyl or ethyl) were examined. Furthermore, the impacts of polyol molecular weight and the ratio of isocyanate to hydroxyl (NCO/OH) on viscosity of SPUR were studied.

## 2. Experimental

### 2.1. Materials

#### 2.1.1. Materials for the Synthesis of SPUR Polymers

Polypropylene glycols PPG 2000 (OH value = 56 mg KOH/g, molecular weight (*M*_w_) = 2000 g/mol, 98%), PPG 4000 (OH value = 26.5–29.5 mg KOH/g, *M*_w_ = 4000 g/mol, 98%), PPG 8200 (OH value = 13.5–15.5 mg KOH/g, *M*_w_ = 8200 g/mol, 98%), isophorone diisocyanate (IPDI, 98%), and vinyl trimethoxysilane (VTMO,98%) were obtained from Sigma-Aldrich, Espoo, Finland. Metal catalysts dioctyltin dilaurate (DOTL) (metal content = 15.5%–17.0%) and bismuth carboxylate (metal content = 19.0–21.0%) were obtained from TIB chemicals, Mannheim, Germany. The general information of secondary aminoalkoxysilanes is shown in Table 1. Prior to use, polyols and glassware were dried at 60 °C overnight using reduced pressure.

#### 2.1.2. Materials for SPUR Formulations

Surface coated and precipitated calcium carbonate (Sigma-aldrich, Espoo, Finland, ≥99.9%), alkyl sulphonic ester of phenol (Sigma-Aldrich, Espoo, Finland), vinyltrimethoxysilane (Sigma-Aldrich, Espoo, Finland, 98%) and 3-aminopropyltrimethoxysilane (Sigma-Aldrich, Espoo, Finland, 97%) were utilized as additives in SPUR formulations.

### 2.2. Synthesis of SPUR Prepolymer

The SPUR prepolymers were prepared via a two-stage process; the synthesis of NCO-terminated urethane prepolymers was followed by silane end-capping. The synthesis reactions were carried out in a three-neck round bottom flask equipped with a mechanical stirrer, a dropping funnel and a water condenser connected to nitrogen inlet. Inert atmosphere was maintained throughout the whole process to ensure absence of moisture.

#### 2.2.1. Synthesis of NCO-Terminated Urethane Prepolymer

NCO-terminated PUR was prepared in a one-shot process. The synthetic one-shot procedure is exemplified by the synthesis of SPUR6. Thus, SPUR6 was prepared as follows: 22.2 g (0.1 mol) IPDI, 200 g (0.05 mol) PPG 4000 and 0.3 g catalyst Bi2 (0.13 weight%, metal content around 0.06 g) were added into the reactor under a blanket of nitrogen. The mixture was then heated up to 70 °C under vigorous stirring of 800–1000 rpm. Temperature in the flask was kept constantly below 80 °C to avoid undesirable side reactions.

The reaction conversion was followed by attenuated total reflectance-Fourier transform infrared spectroscopy (ATR-FTIR). During the reaction progress, the relative intensity of urethane C=O band at 1680–1750 cm^−1^ increased, whereas the N=C=O stretching band at 2270 cm^−1^ decreased. The reaction was judged to be completed when no relative changes in intensities were recorded.

#### 2.2.2. Silane End-Capping

After cooling down the reaction flask to below 60 °C, 11.07 g (0.05 mol) secondary amino alkoxysilane S1 was added dropwise into the obtained PUR prepolymer under vigorous stirring. The silane end-capping reaction was monitored by following the increase in the intensity of the urea C=O vibration at 1600–1680 cm^−1^ and the decrease in intensity of the N=C=O stretching band at 2270 cm^−1^ in ATR-FTIR spectra. An absence of absorbance band at 2270 cm^−1^ indicated that free isocyanate had been completely end-capped. Subsequently, 0.3 g VTMO (0.13 weight %) was added as a moisture scavenger to enhance the pot life of the products. SPUR prepolymers were obtained as viscous liquids.

The compositions of different SPUR prepolymers are shown in Table 2. The molar ratio of diisocyanate to polyol (NCO/OH) and the weight ratio of hard segment to soft segment (HS/SS) were calculated according to the methods presented in our previous work [16].

#### 2.2.3. SPUR Formulation

The composition of SPUR is shown in Table 3. The filler, moisture scavenger, plasticizer and adhesion promoter were added to the synthesized SPUR polymer by rapid mixing (2 min). The formulated SPUR are coded as SPUR-F.

#### 2.2.4. Curing of SPUR-F Films

The formulated SPURs were set in dumbbell-shaped PTFE molds to cure at 23 °C and at a relative humidity of 50%. The cure rates of these samples were followed up over 7 days (7d), and the tack-free time was noted. The same specimens were used for mechanical tests.

### 2.3. Characterization Methods

NMR Nuclear magnetic spectroscopy of SPUR prepolymers was carried out using a Bruker 500 MHz spectrometer (Bruker AXS Nordic AB, Solna, Sweden). CDCl_3_ was used as the solvent.

ATR-FTIR Attenuated total reflectance-Fourier transform infrared spectroscopy (IS50 ATR-FTIR instrument from Thermo Scientific, Thermo Fisher Scientific Finland, Vantaa, Finland) was utilized to analyze both SPUR prepolymers and cured SPUR films. Absorbance spectra were collected between 400 and 4000 cm^−1^ at a resolution of 4 cm^−1^.

TGA Thermal stability of SPUR prepolymers and cured SPUR films was investigated by thermogravimetric analysis (TGA) (TA Instrument SDT Q600, TA instruments Sweden, Sollentuna, Sweden). Samples were analyzed under nitrogen atmosphere (flow rate 100 mL/min) over a temperature range of 25 to 800 °C at a heating rate of 10 °C/min.

Rheology The viscosity of SPUR prepolymers was evaluated by using a rotational rheometer Physica MCR 301 (Anton Paar GmbH, Anton Paar, Graz, Austria). A cone-plate geometry (CP25-1, Anton Paar, Graz, Austria) was used, with a diameter of 25 mm and cone angle of 1 degree. Measurements were done at 23 °C, at shear rates ranging from 1–100 s^−1^.

The viscosities of SPUR-F were measured using a Brookfield viscometer with a spindle to speed ratio of 7/20. Viscosity measurements were performed the same day as the formulations were prepared.

Mechanical strength of the cured SPUR samples after 7 days of curing was measured using a tensile testing instrument (Instron 3345, Instron Europe, Darmstadt, Germany), according to the standard DIN 53504/ ASTM D412/ ISO 37. The crosshead speed was set to 50 mm/min. Tensile results are given as an average of at least five specimens.

## 3. Results and Discussion

### 3.1. NMR

NMR is a sensitive and powerful tool to study the chemical structure of SPUR prepolymers and the reactivity of primary and secondary isocyanates in IPDI, as well as to predict number-average molecular weight of SPUR prepolymers [12,22,23].

**Figure 2:**
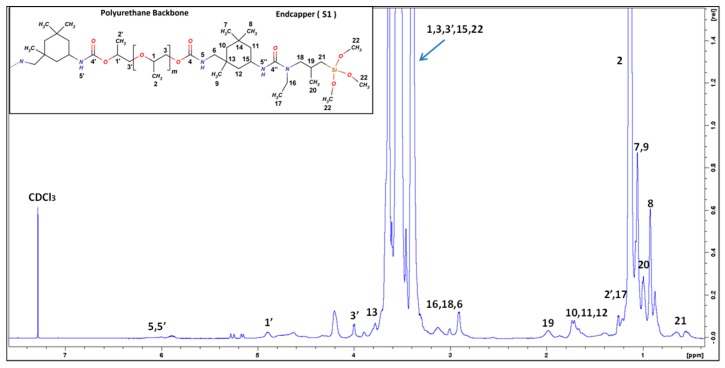
^1^H-NMR spectrum of SPUR 6.

**Figure 3:**
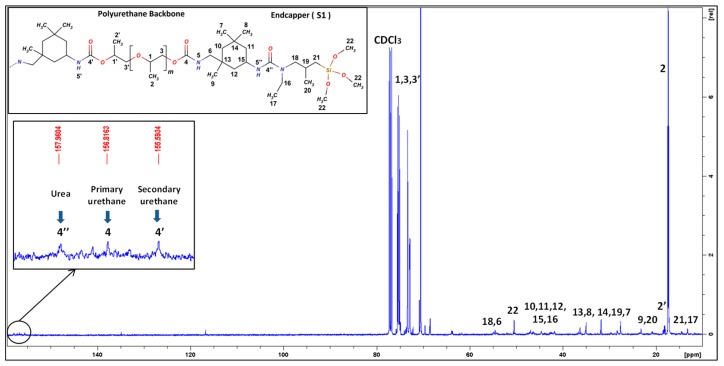
^13^C-NMR spectrum of SPUR 6.

As an example, the ^1^H and ^13^C NMR spectra of SPUR 6 are shown in Figure 2 and Figure 3. The assignments of its characteristic resonance signals were based on the 2D spectra of proton-proton correlation (^1^H-^1^H COSY) and proton-carbon single- and multiple-bond correlation (^1^H-^13^C HSQC and HMBC). The protons of α-methylenes next to alkoxysilanes (H21) show a resonance signal at around 0.5–0.6 ppm, except for SPUR 10 (S5), which has a higher chemical shift of around 2.5 ppm due to the effect of the nearby nitrogen atom. The methine (H1’) of PPG close to urethane shows a characteristic chemical shift at 4.9 ppm, which was used for the estimation of the number-average molecular weight (*M*_n_) of SPUR prepolymers. *M*_n_ calculation was accomplished by calibrating the integral of H21 to be 4, which refers to a SPUR prepolymer chain with two alkoxysilane terminals. Thus, the integral of H1’ indicates the amount of urethane units in a SPUR prepolymer chain. In this way, it was deduced that there are, on average, 5.0 urethane units per chain in SPUR 1 (1.5:1), 1.2 urethane units in SPUR 2 (2:1) and 0.6 urethane units in SPUR 3 (2.8:1). The polymer chain length increases as the NCO/OH decreases, which is in accordance with previous findings [17].

**Figure 4:**
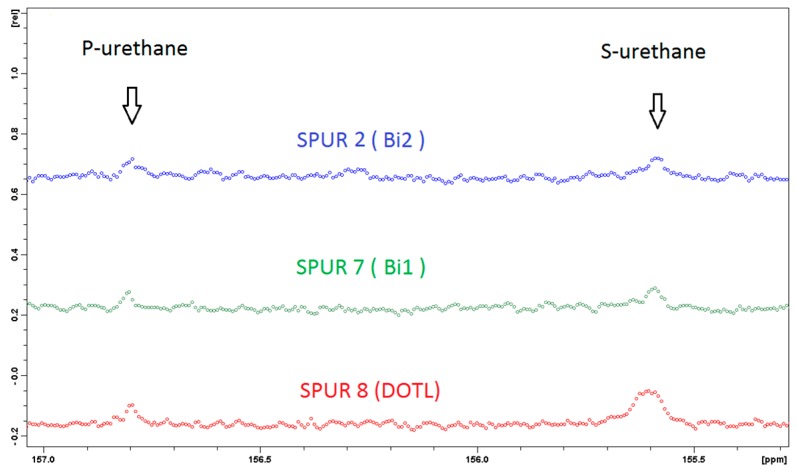
The expanded ^13^C-NMR spectra (155–157 ppm) for samples with different catalysts.

IPDI is an asymmetric cycloaliphatic diisocyanate, containing primary isocyanate (p-NCO) (bonded through a primary carbon) and secondary isocyanate (s-NCO) (bonded directly to the cycloaliphatic ring), which may exhibit different reactivity towards active hydrogen compounds. Reactivity differences in the presence of catalysts can be studied by investigating the carbon resonances in the urethane region from 155 to 157 ppm, as the primary urethane (P-UR) and secondary urethane (S-UR) have clearly different resonance frequencies in ^13^C-NMR spectroscopy [14,22,23]. The two peaks at 155.6 and 156.8 ppm in Figure 3 and Figure 4 were assigned to the carbonyls in S-UR and P-UR, respectively. SPUR 8 catalyzed by DOTL has a higher relative peak intensity at S-UR (155.6 ppm) than the one at P-UR (156.8 ppm), which indicates that DOTL is more selective towards the secondary isocyanate. In comparison, the samples with bismuth catalysts show similar relative intensities of peaks in the S-UR and P-UR regions.

### 3.2. ATR-FTIR Characterization

ATR-FTIR measurements were used to monitor the progress of synthetic reactions and to characterize the structure of SPUR prepolymers, as well as curing of SPUR films [9,13,15,24]. In particular, it provides a convenient and effective way to characterize the organization and association of polymer chains, in terms of the types and extent of hydrogen bonding interactions [16]. Meanwhile, the catalyst activity and silane reactivity can be evaluated. In addition, the relative reactivity of the primary isocyanate (p-NCO) and secondary isocyanate (s-NCO) in IPDI for different catalysts can be studied [23].

#### 3.2.1. Reaction and Cure Progress

##### Reaction Progress

**Figure 5:**
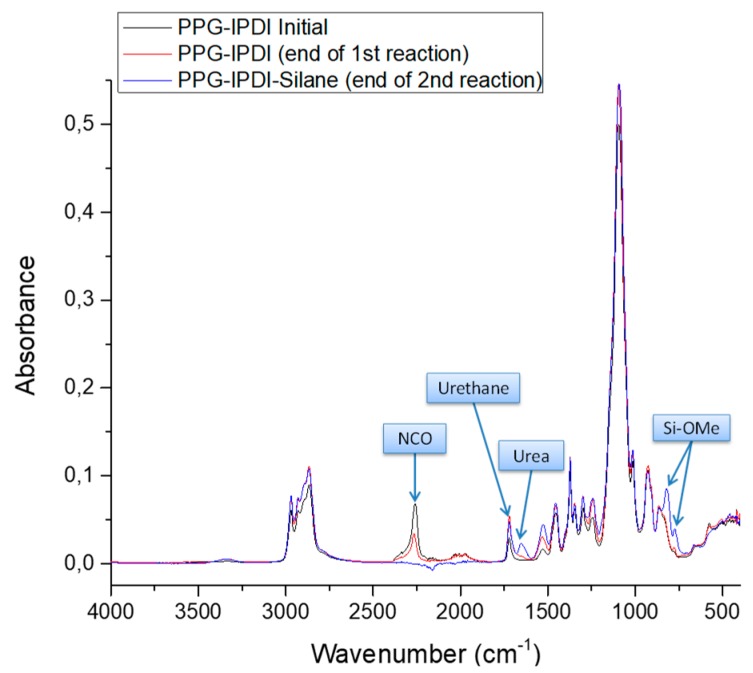
The comparison of ATR-FTIR spectra of SPUR 8 taken at different reaction stages.

The ATR-FTIR spectra of SPUR 8 taken at different reaction stages are compared in Figure 5. The characteristic functional groups are assigned in Table 4. During the 1st stage reaction between PPG (OH) and IPDI (NCO), the intensity of the NCO absorbance peak at 2260 cm^−1^ decreased and the intensity of the absorbance peak of carboxyl in the urethane linkage at 1750–1700 cm^−1^ increased. No changes of these peaks marked the end of the 1st stage reaction. In the 2nd stage, the reaction between secondary aminosilane and isocyanate end groups resulted in new absorbance peaks at 1680–1600 cm^−1^ (carboxyl in urea linkages) and 817–774 cm^−1^ (methoxysilane Si–OMe). The intensity of NCO absorbance peak continuously decreased as the 2nd stage reaction proceeded. The disappearance of the NCO absorbance peak indicated the end of the 2nd stage reaction.

##### The Progress of Curing

**Figure 6:**
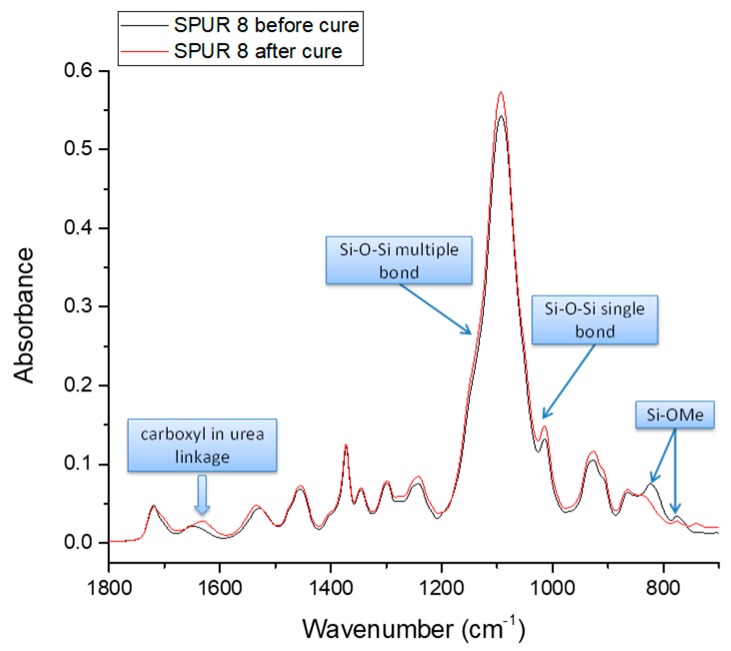
The comparison of ATR-FTIR spectra of SPUR 8 before and after cure.

The ATR-FTIR spectra of SPUR 8 before and after cure are compared in Figure 6. The curing process results in decreased intensities of Si–OMe absorbance peaks, which are clearly observed at 774 and 817 cm^−1^, and the appearance of siloxane Si–O–Si absorbance peaks (single siloxane bond at 1040–1060 cm^−1^ and multiple siloxane bonds at 1120–1140 cm^−1^) [9,13,24]. However, the siloxane absorbance peaks are barely noticed, as they overlap with strong signals of C–O–C in polyurethane backbone at 1100 cm^−1^. Another significant finding is that the absorbance peak of carboxyl in urea linkages (1680–1600 cm^−1^) shifts to a lower wavenumber upon curing, which indicates that, irrespective of the crosslinking reactions, the hydrogen bonding ability of urea linkages increases upon siloxane formation.

#### 3.2.2. The Reactivities of NCOs in IPDI

**Figure 7:**
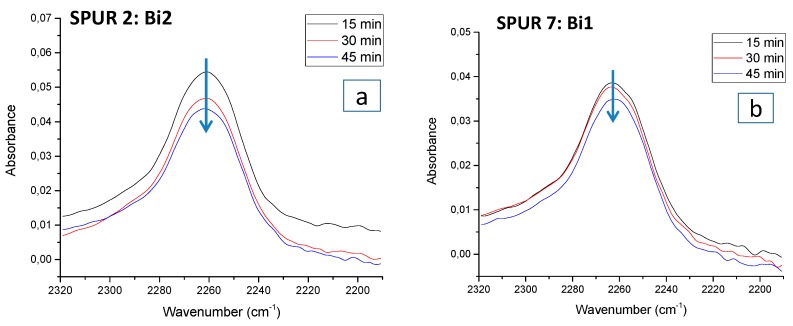

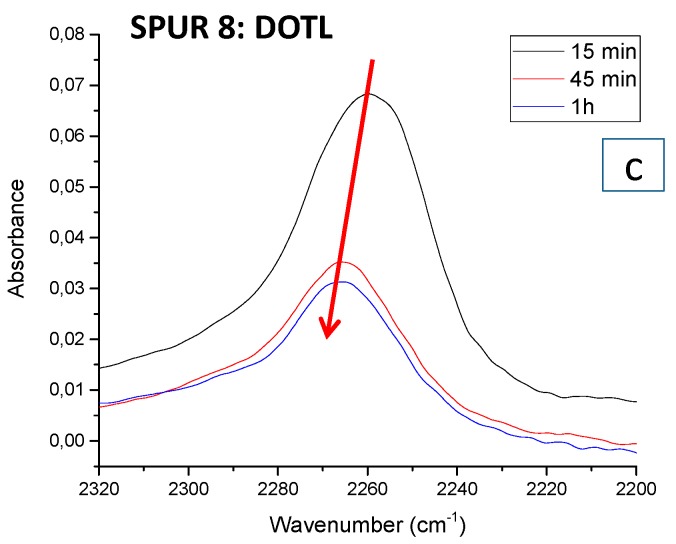
ATR-FTIR spectra in the isocyanate stretching region of samples taken at different reaction times during 1st stage reaction for different catalyst systems: (**a**) SPUR 2 (Bi2); (**b)** SPUR 7 (Bi1); (**c**) SPUR 8 (DOTL).

The effect of catalyst on the reactivity of isocyanates in IPDI can be investigated using FTIR. Generally, isocyanate vibrations give rise to a single broad band centered at around 2260–2255 cm^−1^, which is a combined signal from p-NCO and s-NCO difficult to be distinguished. However, the shape of the peak can change, if the p- and s-NCO have different reactivity. According to Figure 7, isocyanate peaks of SPUR 6 (Bi2) and SPUR 7 (Bi1) had no significant change in peak position during the reaction progress, which indicates that the reactivity of p- and s-NCO was similar in catalyst systems based on bismuth carboxylates (Bi1 and Bi2). On the contrary, in the DOTL system, it was obvious that the isocyanate peak shifted to a higher wavenumber. This strongly indicates that the reactivity of p- and s-NCO was different in the DOTL system. Since the p-NCO band has been reported to appear at a slightly higher wavenumber than the s-NCO in the FTIR spectrum [23], it is suggested that the reactivity of s-NCO is higher than that of p-NCO in the DOTL catalyzed system. This finding confirms the NMR results, and it is also in line with the previous findings suggesting a higher reactivity of s-NCO towards hydroxyls in tin-catalyzed reactions [14,23,25]. Thus, based on this observation, we can conclude that the reactivity of p- and s-NCO towards hydroxyl groups is highly dependent on the catalyst system.

#### 3.2.3. Catalyst Activity and Silane Reactivity

##### Catalyst Activity

Catalysts play a dominant role in accelerating the isocyanate-hydroxyl reaction (PUR prepolymer formation), particularly in the presence of IPDI with a low reactivity. The activities of different organometallic catalysts were evaluated by simply monitoring the time required for reaction to go to completion by ATR-FTIR.

Comparing samples prepared under similar conditions in the presence of 0.1 wt % of different types of catalysts, the times for their urethane prepolymer formations are shown in Table 5.

Irrespective of the catalyst type, substantial urethane formation was detected after 15 min of mixing at 70 °C. According to the ATR-FTIR results, no significant differences in urethane signals of SPUR 6 (catalyzed by Bi2) and SPUR 7 (catalyzed by Bi1) could be observed after 45 min of reaction, which indicated that these two bismuth carboxylates have similar activities, while for sample SPUR 8 catalyzed by DOTL it took 60 min to complete the reaction. This suggests that bismuth carboxylates have an initially higher catalysis activity than DOTL [5,26,27]. Previous studies have shown that tin catalyst (DBTDL) can form complexes with both isocyanate and hydroxyl moieties that in turn serve as intermediates, which convert into urethane in the subsequent rate-determining step. Consequently, the catalyst efficacy is also dependent on whether a one-shot or two-stage process is utilized [28,29]. DBTDL addition in a mixture of polyol and isocyanates (one-shot process) results in a more rapid formation of polyurethane than when adding DBTDL catalyst in a two-stage process [29] This is one reason that we synthesized PUR prepolymers in a one-shot process. DOTL has a similar chemical structure as DBTDL, and thus a similar catalytic mechanism is to be expected.

##### Silane Reactivity

The effect of the chemical structure of silanes, in terms of substitution pattern (alicyclic, cyclic or aromatic), spacer (α or γ) and alkoxyl (methoxy or ethoxy) groups, on their reactivity in 2nd stage reaction was studied. By virtue of the convenience of the ATR-FTIR method, the reactivity of different silanes was evaluated by recording the time required for completing the reaction. The reaction was considered to be completed when –NCO signal could not be detected in the ATR-FTIR spectra.

Five samples, denoted SPUR 2, 6, 9, 10 and 11, were synthesized using different types of silanes, and the end-capping time for the formulations is shown in Table 6.

Aminosilanes S1, S3, S4 and S5 were effective end-cappers, as they were able to completely end-cap the NCO-terminated prepolymers in five minutes. S2 had considerably lower reactivity. Free isocyanate groups were still detectable in SPUR 9 after two hours of reaction. The possible reason to the low reactivity of S2 could be the aromatic ring reduces nucleophilicity of amine.

#### 3.2.4. Polymer Structure

**Figure 8:**
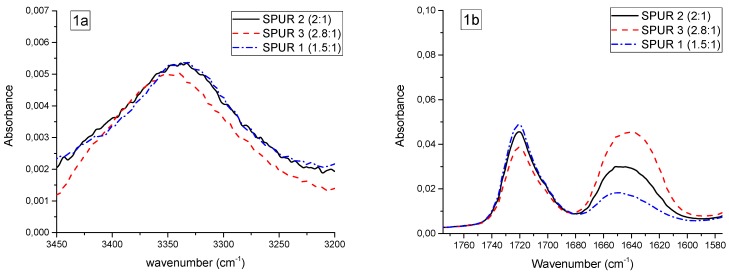

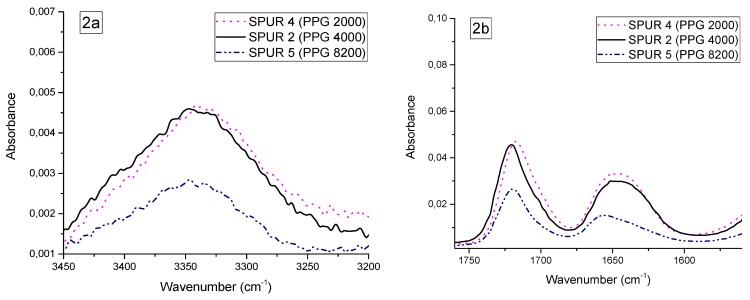
The ATR-FTIR spectral comparison of relevant samples with different variables in N–H stretching region (3450–3200 cm^−1^) (left) and C=O stretching region (1760–1600 cm^−1^) (right). (**1a**,**b**): Impact of NCO/OH ratio; (**2a**,**b**): Impact of PPG chain length.

The mechanical and physical properties of polyurethanes are largely controlled by their dual-phase structure which stems from the extent and patterns of hydrogen bonding interactions. The extent of hydrogen bonding can be investigated by studying the urethane N–H stretching region (3450–3200 cm^−1^) and C=O stretching regions (amide I: 1760–1600 cm^−1^) in ATR-FTIR spectrum [16]. As the polymer structure is primarily influenced by NCO/OH ratio and PPG chain length, their impacts were elaborated by comparing ATR-FTIR spectra of relevant SPUR prepolymers in these stretching regions, as shown in Figure 8.

##### Molar Ratio of NCO/OH

According to Figure 8(1a,1b), NCO/OH ratio had little impact on the N–H (3450–3200 cm^−1^) and urethane C=O (1760–1680 cm^−1^) absorbance peaks. Meanwhile, the recorded slight decrease in the intensity of urethane absorbance peaks as the NCO/OH ratio increases can be attributed to the decrease in PUR chain length. Not surprisingly, it was observed that urea C=O (1680–1600 cm^−1^) absorbance increased as the NCO/OH ratio increased. Besides, the N–H peak of SPUR 3 (NCO/OH = 2.8:1) slightly shifted to a higher wavenumber, which also indicates a higher urea content [25].

##### PPG Molecular Weight

In Figure 8(2a,2b), it can be observed that N–H (3450–3200 cm^−1^) and urethane C=O (1760–1680 cm^−1^) absorbance peaks slightly shifted to a higher wavenumber as PPG molecular weight increased. In particular, SPUR 5 (PPG 8200), with a low content of hard segments (HS/SS ratio in Table 3), has less hydrogen bonding interactions (urea C=O shifted to a higher wavenumber).

### 3.3. Rheology of SPUR Prepolymers

The rheology of SPUR prepolymers was investigated at low and high shear rates in order to simulate the deformation processes to which the prepolymers are subjected to during storage and application processes. The low shear rate condition ($\dot{\gamma }$ = 1 s^−1^) simulates samples at steady state, giving important information closely related to the average molecular weight and polymer chain interactions. On the other hand, the high shear rate simulates application conditions of SPUR polymers.

The SPUR prepolymers showed considerable shear-thinning behavior (see Table 7), and the decrease in viscosities at higher shear rates have been attributed to gradual breakdown of secondary intramolecular forces [18]. By comparing SPUR 2 (PPG 4000), SPUR 4 (PPG 2000) and SPUR 5 (PPG 8200), it can be noted that viscosities of SPUR prepolymers increased with PPG chain length at a shear rate of 1 s^−1^, which indicates that PPG chain length plays a dominant role in the evolution of viscosity at steady state. As could be anticipated, shear thinning was more pronounced for SPUR 5 (PPG 8200) than for the other samples, as the weaker secondary intermolecular forces (less hydrogen bonding interactions) in the SPUR 5 (PPG 8200) sample are insufficient to hold polymer chains together at a high shear rate. In contrast, SPUR 2 (PPG 2000) exhibited only a moderate shear thinning behavior, as the relatively high content of hydrogen bonding interactions contribute to the ability to withstand increasing shear rates.

The viscosities of SPUR prepolymers SPUR 1 (1.5:1), SPUR 2 (2:1) and SPUR 3 (2.8:1) decreased as the NCO/OH ratios increased, which is attributed to a decreasing polyurethane chain length. This is in line with earlier studies [17]. The shear thinning is less pronounced for SPUR 3 with the highest NCO/OH ratio, because of its substantial hard segment content (urea) effectively reducing chain mobility via hydrogen bonding. Among the samples containing different silanes, SPUR-11 (S3) exhibited considerably higher viscosity than other samples, which could be attributed to the bis-(trimethoxysilylpropyl) amine structure [30,31]. Comparing samples with different catalysts, samples with DOTL and Bi2 exhibited considerably lower viscosities.

The viscosities of formulated SPUR were measured by a Brookfield viscometer, in order to evaluate their open time and viability as a SPUR in practice. When compared with their prepolymer counterparts, it was generally observed that by appropriate formulation design, one can adjust the viscosities of SPUR polymers to a reasonably low level by the addition of plasticizers. 

### 3.4. Cure Rate of Formulated SPUR

#### 3.4.1. Effect of Catalyst

**Figure 9:**
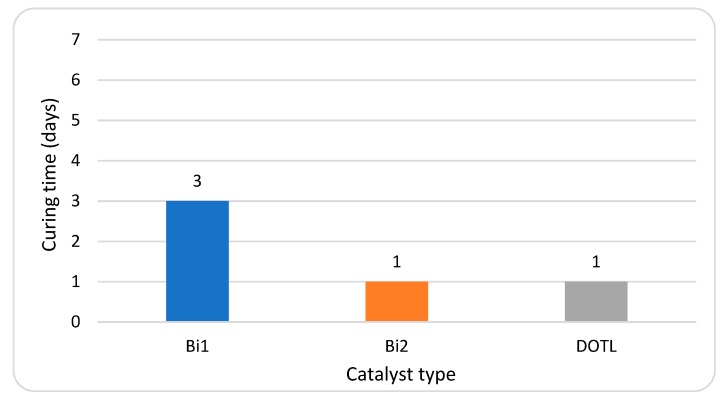
Effect of catalysts on cure time of formulated SPUR samples.

The cure times of formulated samples prepared with different types of catalysts are compared in Figure 9. The sample with bismuth carboxylate catalyst Bi1 had the longest cure time (3 days), which can partly depend on it having the lowest metal content (15–16.5%) among all the samples. Bismuth carboxylate Bi2, with a high metal content (19–21%), induced curing as fast as DOTL (15.5–17.0%) (1 day), and it also exhibited high catalytic activity in the silanol-water reaction. These results can be explained by the fact that bismuth with a lower Lewis acidity in comparison to a tin catalyst has a tendency to lose activity during the curing process, and therefore an initially higher metal content is needed for a bismuth catalyst than for a tin catalyst [5].

#### 3.4.2. Effect of Silane

**Figure 10:**
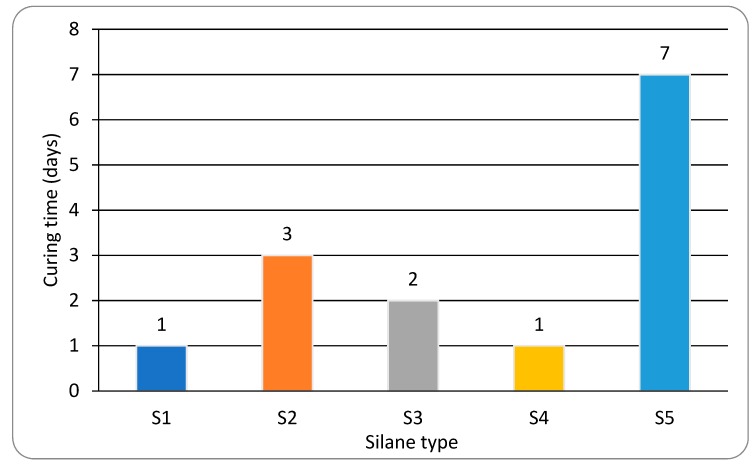
Effect of silanes on cure time of formulated SPUR samples.

In the presence of 0.1% bismuth carboxylate Bi2, the cure times of formulated samples containing different types of silanes were compared (Figure 10). Formulated SPUR samples containing S1 and S4 showed the fastest cure (1 day). In comparison, slightly longer cure times (2d or 3d) were required for samples containing S3. The sample containing S5 had the slowest cure, and even after 7d bulk curing was incomplete. A soft skin formation was observed in the sample with S5, which indicates that the α-linked nitrogen in the silane induced such a fast cure on the film surface that a complete through-cure underneath was delayed.

### 3.5. Mechanical Properties of Cured SPURs

Mechanical properties of cured SPUR, including tensile strength and elongation at break, are shown in Table 8, Table 9, Table 10 and Table 11.

By comparing SPUR-4-F (PPG 2000), SPUR-2-F (PPG 4000) and SPUR-5-F (PPG 8200) (Table 8), one can see that the elongation increased as PPG molecular weight increased due to enhanced flexibility of the polymer chains. However, it was surprisingly observed that the tensile strength also slightly increased with an increase in PPG molecular weight. Despite that SPUR-5-F exhibited good mechanical strength, its high viscosity (Table 7) makes it less favorable. 

Mechanical test results of samples with different NCO/OH are compared in Table 9. Tensile strength of SPUR-1-F (NCO/OH = 1.5) was significantly lower than for SPUR-2-F (NCO/OH = 2) and SPUR-3-F (NCO/OH = 2.8), which indicated that a low NCO/OH ratio gave a weak bondline. SPUR-2-F and SPUR-3-F have high NCO/OH ratio, and thereby an increased content of silane and urea linkages, as well as greater hydrogen bonding interactions, which contribute to greater crosslink density and enhanced tensile strength. As expected, the elongation decreased as a function of NCO/OH, due to the increased stiffness and reduced flexibility.

Similar tensile strength results were observed with different catalysts (Table 11), but the sample prepared with DOTL showed slightly lower elongation than the other samples. The mechanical strengths of samples containing different silanes were also compared (Table 10). The best mechanical strength results were observed for SPUR-6-F with silane S1, which simultaneously exhibited high tensile strength and elongation. Comparable good results were obtained for SPUR-2-F with S4. The lowest tensile strength was observed for SPUR-11-F (S3).

## 4. Conclusions

A series of moisture-curable silane-terminated polyurethanes (SPUR) was prepared, and the effects of polyol (PPG) molecular weight, NCO/OH ratio and the types of catalyst and aminosilane used in the synthesis of SPUR polymers were investigated. SPUR polymers of significantly lower viscosity were obtained by using PPG of molecular weight 2000 and 4000 g/mol in comparison to 8200 g/mol. Furthermore, increasing the NCO/OH ratio was found to lead to SPUR of favorably low viscosity. Bismuth carboxylates Bi1 and Bi2 were effective catalysts in the synthesis of SPUR prepolymers, even superior to the tin catalyst DOTL. Most of the tested secondary aminosilanes were found to be effective end-cappers for the NCO-terminated polyurethanes, since complete end-capping was achieved according to the FTIR studies.

The curing behavior of SPUR was found to be dependent on the silane and catalyst type, and a 1-day curing time was established to be sufficient for the SPURs catalyzed by the bismuth carboxylate of a high metal content (Bi2) and the tin catalyst DOTL when using either aminosilane S1 or S4. (Noteworthy is that toxic methanol is released from the SPURs during moisture curing). Mechanical properties of the cured SPUR samples, in terms of tensile strength and elongation, are closely related to the flexibility of polymer chains, the content of silane, and hard segments, as well as the extent of hydrogen bonding. An optimal performance with balanced flexibility and toughness was obtained with a SPUR formulation containing the bismuth carboxylate catalyst Bi2, medium long polymer chains (PPG 4000 and NCO/OH = 2) and silane end-cappers S1 or S4.

The presented results give an insight into the effects of bismuth catalysts and the polymer composition on the performance of SPURs. It gives good guidelines for selecting raw materials for the synthesis of low viscous organotin-free high-performance moisture-curable silane-terminated polyurethanes.

## Figures and Tables

**Table 1:** General information of secondary aminoalkoxysilanes.

Silane Code	Chemical Name	Purity	Chemical Structure	Supplier
S1	*N*-ethyl-aminoisobutyl-trimethoxysilane	98%	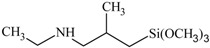	ABCR (Germany)
S2	*N*-phenyl-aminopropyl-trimethoxysilane	98%	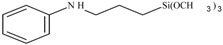	ABCR (Germany)
S3	Bis((3-trimethoxysilyl)-propyl)amine	98%	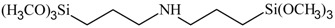	ABCR (Germany)
S4	*N*-butyl-aminopropyl-trimethoxysilane	98%	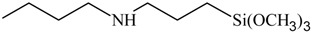	ABCR (Germany)
S5	*N*-cyclohexyl-aminomethyl-triethyoxysilane	98%	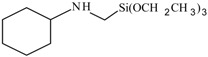	ABCR (Germany)

**Table 2:** The sample codes and compositions of the synthesized SPUR prepolymers.

SPUR Sample Code	Polyol Molecular Weight (g/mol)	Diisocyanate	NCO/OH	Catalyst (0.1 wt %)	Secondary Aminosilane	HS/SS (wt %)
SPUR 1	PPG 4000	IPDI	1.5	Bi2	S4	8.35
SPUR 2	PPG 4000	IPDI	2.0	Bi2	S4	11.10
SPUR 3	PPG 4000	IPDI	2.8	Bi2	S4	15.56
SPUR 4	PPG 2000	IPDI	2.0	Bi2	S4	22.20
SPUR 5	PPG 8200	IPDI	2.0	Bi2	S4	5.42
SPUR 6	PPG 4000	IPDI	2.0	Bi2	S1	11.10
SPUR 7	PPG 4000	IPDI	2.0	Bi1	S4	11.10
SPUR 8	PPG 4000	IPDI	2.0	DOTL	S4	11.10
SPUR 9	PPG 4000	IPDI	2.0	Bi2	S2	11.10
SPUR 10	PPG 4000	IPDI	2.0	Bi2	S5	11.10
SPUR 11	PPG 4000	IPDI	2.0	Bi2	S3	11.10

**Table 3:** The composition of SPUR formulation.

Type	Details	Parts by Weight
Polymer	SPUR prepolymer	20.00
Filler	Surface coated and precipitated calcium carbonate	62.00
Plasticizer	Alkyl sulphonic ester of phenol	15.00
Moisture scavenger	Vinyltrimethoxysilane	2.00
Adhesion promoter	3-aminopropyltrimethoxysilane	1.00
Total		100

**Table 4:** The assignments of functional groups of SPUR 8.

Amide Type	Functional Groups	Wavenumber (cm^−1^)
	Urethane N-H stretching	3300–3400
Amide I	Urethane C=O	1750–1700
	Urea C=O	1680–1600
Amide II	N–H in-plane bending, C–N stretching	1500–1600
Amide III	C–N stretching, N-H bending	1200–1400
	PPG C-O-C	1100
	Si-OMe rocking	1194
	Si-OMe	2840, 1085, 863, 817, 774

**Table 5:** The evaluation of different catalyst activities.

SPUR Sample Code	Catalyst (Metal Content)	Time for Urethane Prepolymer Formation (min)	Amount of Silane
SPUR 2	Bi2 (19.0%–21.0%)	45	S ^a^
SPUR 7	Bi1 (15.0%–16.5%)	45	S
SPUR 8	DOTL (15.5%–17.0%)	60	S

^a^ S refers to stoichiometric.

**Table 6:** The evaluation of reactivity of different silanes.

SPUR Sample Code	Silane	Observed End-Capping Time (min)
SPUR 2	S4	5
SPUR 6	S1	5
SPUR 9	S2	>120
SPUR 10	S5	5
SPUR 11	S3	5

**Table 7:** Viscosities of SPUR prepolymers and their formulations at various shear rates.

Sample Code	Viscosity of SPUR Prepolymers (mPa·s) at Different Shear Rates		Viscosity of Formulated SPUR/mPa·s
	$\dot{\gamma }=1\;s-1$	$\dot{\gamma }=100\;s-1$	
SPUR-1	119,000	38,500	83,000
SPUR-2	45,900	20,000	59,300
SPUR-3	21,900	12,900	49,800
SPUR-4	33,000	32,800	52,400
SPUR-5	150,000	48,800	86,800
SPUR-6	30,300	12,000	58,700
SPUR-7	187,000	65,800	62,800
SPUR-8	56,400	20,700	44,800
SPUR-9	35,800	16,500	73,000
SPUR-10	96,000	37,400	59,000
SPUR-11	217,000	64,000	79,000

**Table 8:** The mechanical test results of cured SPUR with different PPG chain length.

SPUR Sample Code*	Variables	Tensile Strength (N/mm^2^)	Elongation at Break (%)
SPUR-4-F	PPG 2000	2.00	17.82
SPUR-2-F	PPG 4000	2.06	43.64
SPUR-5-F	PPG 8200	2.37	67.95

**Table 9:** The mechanical test results of cured SPUR with different NCO/OH ratios.

SPUR Sample Code	Variables	Tensile Strength (N/mm^2^)	Elongation at Break (%)
SPUR-1-F	NCO/OH = 1.5	1.56	71.35
SPUR-2-F	NCO/OH = 2	2.06	43.64
SPUR-3-F	NCO/OH = 2.8	2.05	21.79

**Table 10:** The mechanical test results of cured SPUR with different silanes.

SPUR Sample Code	Variables	Tensile Strength (N/mm^2^)	Elongation at Break (%)
SPUR-2-F	S4	2.06	43.64
SPUR-11-F	S3	1.47	43,22
SPUR-6-F	S1	2.23	54.51

**Table 11:** The mechanical test results of cured SPUR with different catalysts.

SPUR Sample Code	Variables	Tensile Strength (N/mm^2^)	Elongation at Break (%)
SPUR-2-F	Bi2	2.06	43.64
SPUR-7-F	Bi1	2.13	57.97
SPUR-8-F	DOTL	2.07	34.06

Formulated SPUR samples X were abbreviated as SPUR-X-F: X refers to the code; F is the abbreviation for formulated.
